# Neuroendocrine Carcinoma of the Esophagus With Liver Metastasis: A Case Report

**DOI:** 10.7759/cureus.28842

**Published:** 2022-09-06

**Authors:** Mallorie Vest, Deesha Shah, Mahmoud Nassar, Negar Niknam

**Affiliations:** 1 Internal Medicine, Icahn School of Medicine at Mount Sinai/NYC Health+Hospitals Queens, New York City, USA; 2 Gastroenterology, Icahn School of Medicine at Mount Sinai/NYC Health+Hospitals Queens, New York City, USA

**Keywords:** endoscopic ultrasound (eus), neuroendocrine tumor, rare cancer, neuroendocrine carcinoma of esophagus, neuroendocrine carcinoma

## Abstract

Neuroendocrine carcinoma (NEC) of the esophagus is a rare and aggressive malignancy. It is challenging to manage NEC due to its rarity. NEC may be asymptomatic or present with various symptoms such as dysphagia, abdominal discomfort, weight loss, melena, hot flushes, or diarrhea. We present the case of a 55-year-old male with a large cell neuroendocrine carcinoma of the esophagus. His aggressive and rapid progression of neuroendocrine carcinoma of the esophagus resulted in a poor clinical outcome.

## Introduction

Neuroendocrine carcinoma (NEC) of the esophagus is a rare and aggressive malignancy with an incidence rate between 0.4% and 2% [1]. NEC primarily occurs in the lower (34.7%) and middle (55.1%) segments of the esophagus [2]. Only a few case studies have examined the clinical complications, prognosis, and medical management of esophageal NEC [3]. There are several macroscopic features of NEC in the gastrointestinal tract, including submucosal growth and ulceration of the tissue [1]. Based on histopathology, the World Health Organization (WHO) has classified digestive system NECs into three categories: low-grade (G1), intermediate-grade (G2), and high-grade (G3) [2]. Merkel cells and stem cells may be possible candidates for the cellular origin of NEC in the esophagus [1].

## Case presentation

We present the case of a 55-year-old Hispanic male with a history of gastroesophageal reflux disease (GERD). He took Protonix 40,mg daily. He was recently been diagnosed with large cell NEC of the esophagus with metastasis to the liver via esophagogastroduodenoscopy (EGD) and endoscopic ultrasound (EUS). The patient presented to the emergency department with worsening right upper quadrant (RUQ) abdominal pain, generalized weakness, and worsening jaundice over the two weeks.

**Figure 1:**
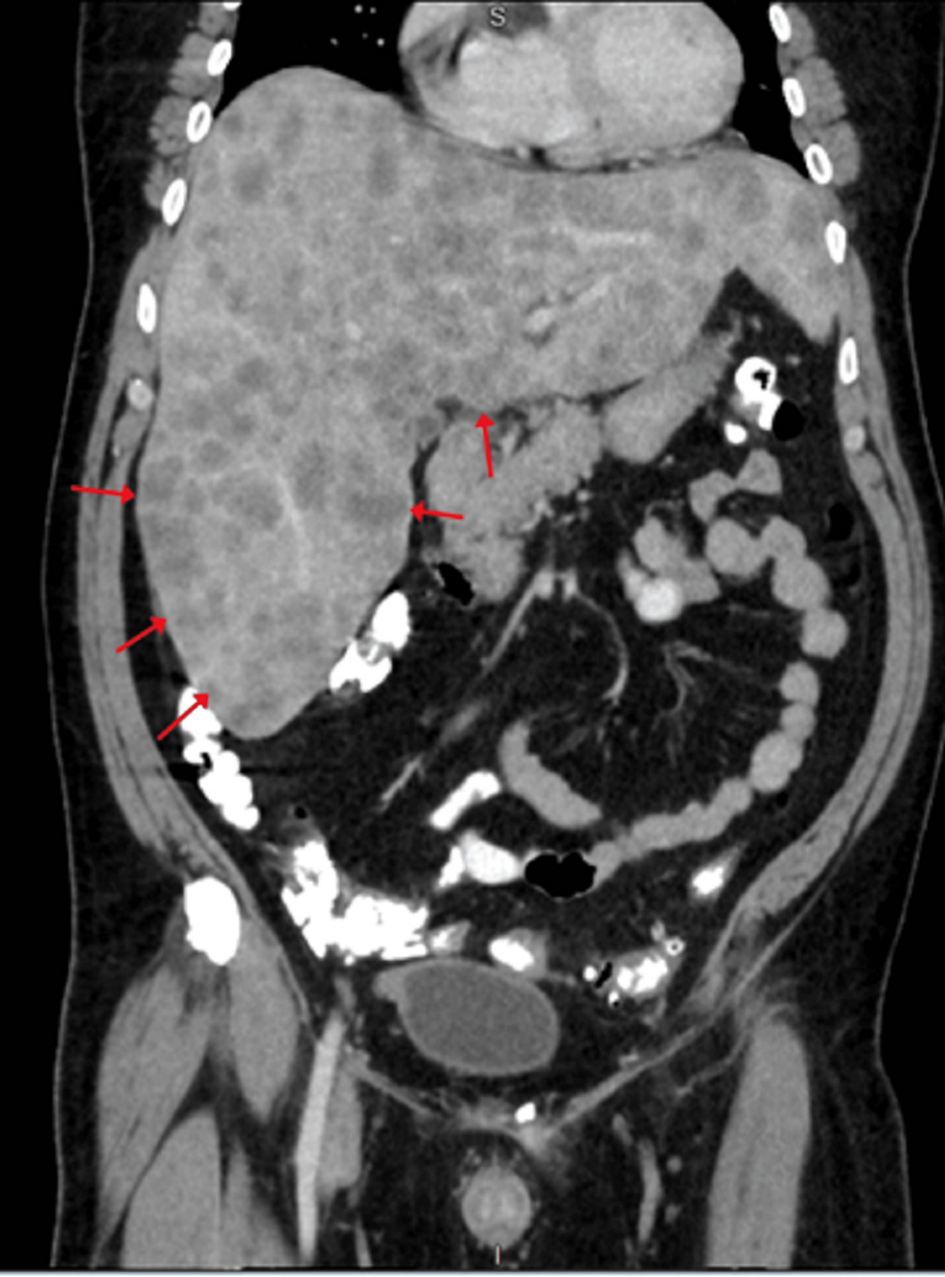
CT Abdomen/Pelvis with contrast showing multiple liver metastases (red arrows)

Physical examination revealed unremarkable vital signs except for mild tachycardia of 105 beats per minute. The patient appeared jaundiced, had right upper quadrant (RUQ) tenderness on palpation, and bilateral 2+ pitting leg edema. The laboratory results are presented in Table 1. A computed tomography (CT) of abdominal/pelvic with contrast revealed hepatomegaly with numerous metastases, retroperitoneal lymphadenopathy, and ascites (Figure 1).

**Table 1:** Laboratory test results with normal value ranges

Lab Test	Result	Normal value range
Potassium (K)	5.4	3.5-5.1 mmoL/L
Blood urea nitrogen (BUN)	65	6-23 mg/dL
Creatinine (Cr)	2.82	0.70-1.20 mg/dL
Direct Bilirubin	>20	0-0.3 mg/dL
Alkaline phosphatase (ALP)	548	40-29 U/L
Aspartate aminotransferase (AST)	539	5-40 U/L
Alanine transaminase (ALT)	192	0-41 U/L

**Figure 2:**
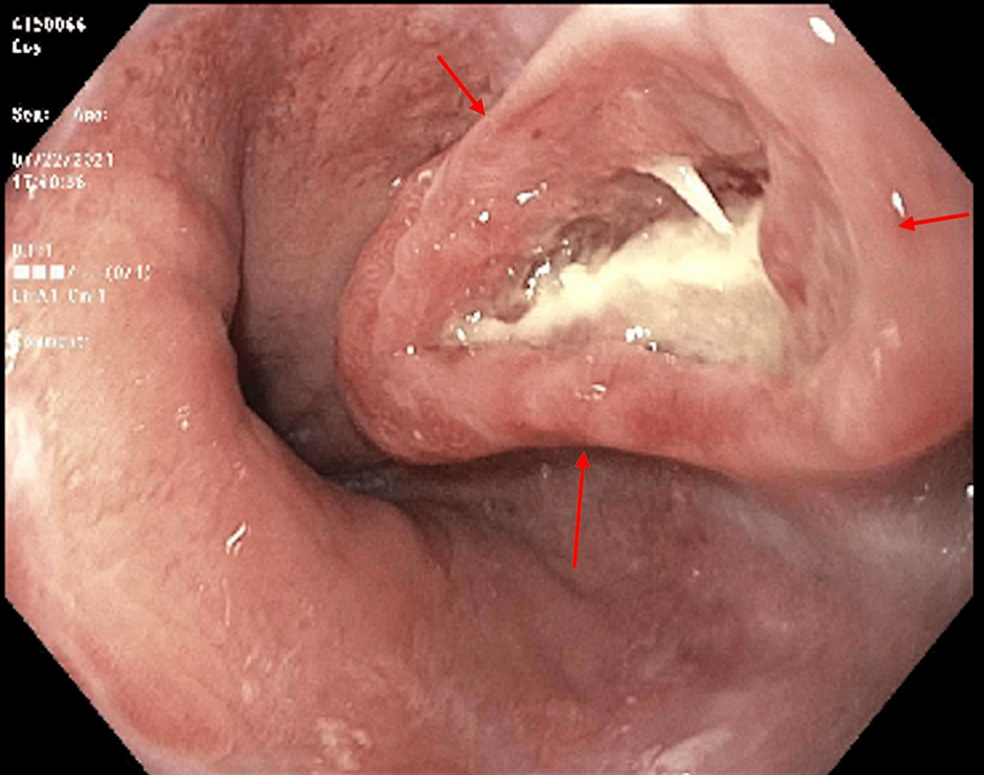
EGD showing lower esophageal mass with deep ulceration (red arrows) EGD: esophagogastroduodenoscopy

**Figure 3:**
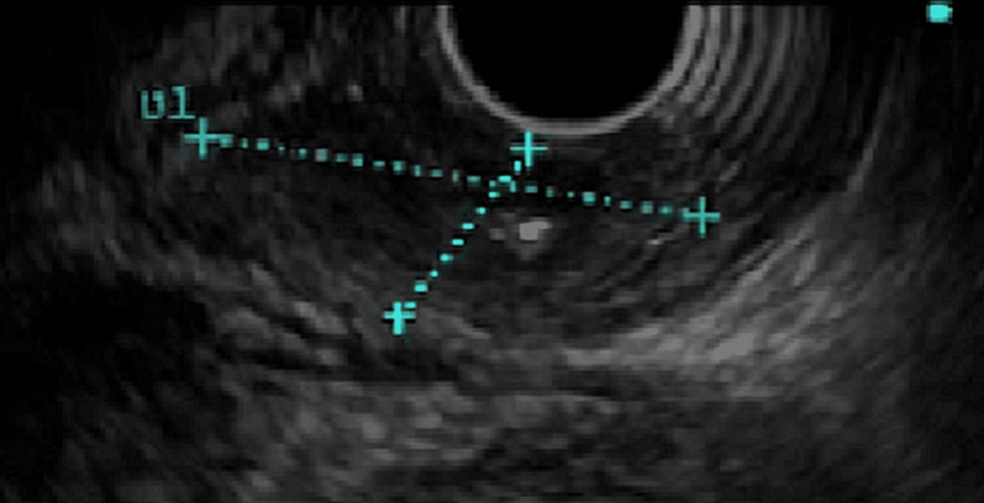
EUS of the esophageal lesion (blue dashed lines) EUS: endoscopic ultrasound.

An EGD with EUS was performed and a non-obstructing lower esophageal mass was found (Figures 2, 3). The mass was biopsied and confirmed as large cell neuroendocrine carcinoma of the esophagus.

Acute renal failure and worsening transaminitis occurred during the patient's current admission. The patient became lethargic and confused as a result of hepatic encephalopathy and hepatorenal syndrome. A nephrologist was consulted, and hemodialysis was not recommended because the patient had a poor prognosis. A chemotherapy treatment could not be initiated due to the advanced stage of the disease. The patient was eventually made a Do-Not-Resuscitate/Do-Not-Intubate (DNR/DNI) following consultation with palliative care. On day 12 of his hospital stay, he became pulseless and was pronounced dead.

## Discussion

Cellular pleiomorphism is the predominant cause of a high-grade and poorly differentiated esophageal NEC. The extremely low incidence, vague symptoms, and absence of a tumor, nodes, and metastases (TNM) classification may contribute to the poor prognosis of NEC [4]. The patient did not qualify for surgical resection or chemotherapy in this case due to metastatic disease and locally advanced presentation [5].

Patients with esophageal NEC may remain asymptomatic (38%) or experience dysphagia (26.9%), abdominal discomfort (19.2%), weight loss (11.5%), melena (7.7%), hot flushes (3.8%), and diarrhea (3.8%) [6]. The current literature has no consensus or standard algorithm for treating esophageal NEC. Endoscopic treatment is appropriate when there are no regional lymph node metastases and the esophageal tumor size ranges between 0.2 and 0.8 cm [7]. The surgical resection selectively targets patients with regional lymph node metastasis or patients with primary tumors [8].

Recurrence of locoregional or metastatic esophageal lesions despite surgical or pharmaceutical treatment worsens the prognosis in patients with esophageal NEC [9]. Somatostatin analogs are warranted for treating hormonal symptoms associated with esophageal NEC, including somatostatin analogue SOM230 and octreotide [10]. The prognosis for patients with pure esophageal NECs is significantly worse than for those with mixed NECs [11].

## Conclusions

NECs of the esophagus are rare, and their pathogenesis, etiology, and prognosis are poorly understood. This makes their management in hospitals extremely challenging. Tumor size, lymph node presence, and metastatic disease are important factors in the selection of appropriate treatment. The histopathological analysis of the large cell subtype of esophageal NECs should be targeted in future studies to improve their prognosis and management.
